# Effects of seasonality and land use on the diversity, relative abundance, and distribution of mosquitoes on St. Kitts, West Indies

**DOI:** 10.1186/s13071-020-04421-7

**Published:** 2020-11-02

**Authors:** Matthew J. Valentine, Brenda Ciraola, Gregory R. Jacobs, Charlie Arnot, Patrick J. Kelly, Courtney C. Murdock

**Affiliations:** 1 One Health Centre for Zoonoses and Tropical Veterinary Medicine, Ross University School of Veterinary Medicine, Island Main Road, West Farm, Basseterre, Saint Kitts and Nevis; 2 Odum School of Ecology, University of Georgia, Athens, GA 30602 USA; 3 River Basin Center, Odum School of Ecology, University of Georgia, Athens, Ga 30602 USA; 4 CNWA Consulting, Basseterre, Saint Kitts and Nevis; 5 Department of Clinical Sciences, Ross University School of Veterinary Medicine, Island Main Road, West Farm, Basseterre, Saint Kitts and Nevis; 6 Department of Infectious Diseases, College of Veterinary Medicine, University of Georgia, Athens, GA 30602 USA; 7 Center for Ecology of Infectious Diseases, Odum School of Ecology, University of Georgia, Athens, GA 30602 USA; 8 Center for Tropical Emerging and Global Diseases, University of Georgia, Athens, GA 30602 USA; 9 Center for Vaccines and Immunology, College of Veterinary Medicine, University of Georgia, Athens, GA 30602 USA; 10 Department of Entomology, College of Agriculture and Life Sciences, Cornell University, Ithaca, NY 14853 USA

**Keywords:** Caribbean, Land cover, Model, Mosquito, Precipitation, Season, Surveillance

## Abstract

**Background:**

Mosquito surveys that collect local data on mosquito species’ abundances provide baseline data to help understand potential host-pathogen-mosquito relationships, predict disease transmission, and target mosquito control efforts.

**Methods:**

We conducted an adult mosquito survey from November 2017 to March 2019 on St. Kitts, using Biogents Sentinel 2 traps, set monthly and run for 48-h intervals. We collected mosquitoes from a total of 30 sites distributed across agricultural, mangrove, rainforest, scrub and urban land covers. We investigated spatial variation in mosquito species richness across the island using a hierarchical Bayesian multi-species occupancy model. We developed a mixed effects negative binomial regression model to predict the effects of spatial variation in land cover, and seasonal variation in precipitation on observed counts of the most abundant mosquito species observed.

**Results:**

There was high variation among sites in mosquito community structure, and variation in site level richness that correlated with scrub forest, agricultural, and urban land covers. The four most abundant species were *Aedes taeniorhynchus*, *Culex quinquefasciatus*, *Aedes aegpyti* and *Deinocerites magnus*, and their relative abundance varied with season and land cover. *Aedes aegypti* was the most commonly occurring mosquito on the island, with a 90% probability of occurring at between 24 and 30 (median = 26) sites. Mangroves yielded the most mosquitoes, with *Ae. taeniorhynchus, Cx. quinquefasciatus* and *De. magnus* predominating. *Psorophora pygmaea* and *Toxorhynchites guadeloupensis* were only captured in scrub habitat. Capture rates in rainforests were low. Our count models also suggested the extent to which monthly average precipitation influenced counts varied according to species.

**Conclusions:**

There is high seasonality in mosquito abundances, and land cover influences the diversity, distribution, and relative abundance of species on St. Kitts. Further, human-adapted mosquito species (e.g. *Ae. aegypti* and *Cx. quinquefasciatus*) that are known vectors for many human relevant pathogens (e.g. chikungunya, dengue and Zika viruses in the case of *Ae. aegypti*; West Nile, Spondweni, Oropouche virus, and equine encephalitic viruses in the case of *Cx. quinqefasciatus*) are the most wide-spread (across land covers) and the least responsive to seasonal variation in precipitation.
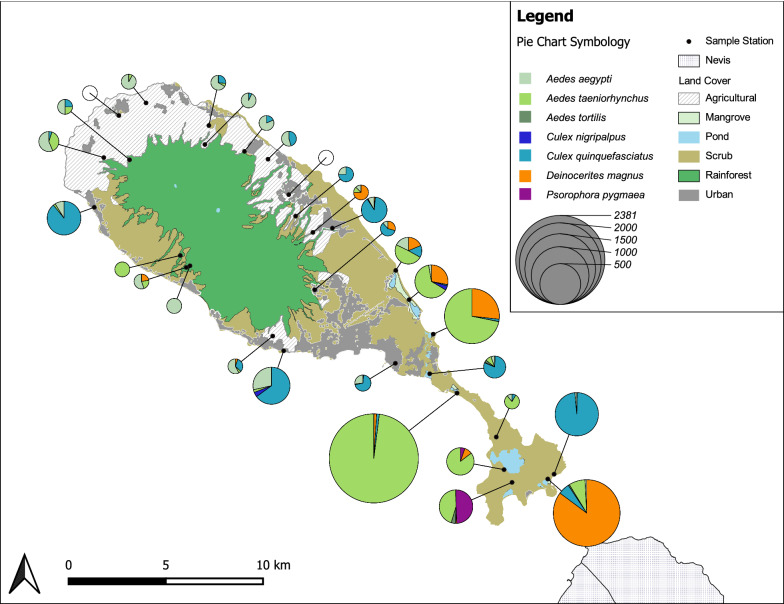

## Background

Mosquitoes are responsible for considerable human and animal suffering and economic losses because of their nuisance value and the diseases of high morbidity and mortality they can transmit [1, 2]. Recent mosquito-borne arboviral pandemics have been able to emerge and spread through human populations in previously unaffected regions, like the Americas [3, 4], due to the widespread presence and abundance of human-adapted mosquito species. Furthermore, mosquito-borne pathogens can become established in new areas if there are suitable animal reservoir populations and mosquito species that can transmit the organisms between these animals (and potentially to humans), as has occurred with yellow fever virus in South America [5, 6].

High quality mosquito surveys are an essential tool for predicting mosquito-borne disease transmission and for mosquito control [7, 8]. Surveys that collect fine resolution local data on mosquito species abundances provide fundamental baseline data on the composition of mosquito communities in a given area, the relative abundances of mosquito species within the community, and how the abundance of species and the composition of mosquito communities change across space and time. The development of population abundance models that leverage count data generated from these surveys, in turn, can be used to predict how mosquito abundances change seasonally and across different land covers. Information of this nature is crucial for describing potential host-pathogen-mosquito relationships in novel transmission foci, accurately predicting disease transmission, and for targeting and assessing the efficacy of mosquito control efforts [8, 9].

St. Kitts is a small tropical island in the Caribbean where local experience shows mosquitoes are very common and their nuisance value high. Outbreaks of chikungunya, dengue, and Zika viruses have recently occurred on the island, which also has a large population of African green monkeys (*Chlorocebus aethiops sabaeus*) that may be involved in arbovirus sylvatic cycles as is the case in Africa [6]. Due to the large numbers of tourists visiting the region each year, islands in the Caribbean like St. Kitts could be a source of mosquitoes, and the pathogens they carry, for transfer into currently naïve areas of the world like the USA [10].

Historically, there has been long standing interest in the mosquito species inhabiting the Caribbean particularly since it was discovered that malarial parasites (*Plasmodium* spp.) and yellow fever virus are transmitted by mosquitoes. Detailed mosquito surveys from the 1970s included several Caribbean islands including St. Kitts and Nevis [11]. The most recent survey on St. Kitts was conducted in 2010 [12] and although this was the most comprehensive survey performed on the island to date, it did not provide data on the how the distribution and relative abundances of mosquito species changes seasonally and with land cover. Mosquitoes were only collected during a single week of the dry and wet seasons and sampling did not include all land covers. As part of an investigation into arboviral sylvatic cycles on St. Kitts, we carried out a comprehensive survey of the mosquito populations across the various land covers on the island on a monthly basis from September 2017 to March 2019. We related mosquito survey data to relevant biological and environmental covariates to assess the influence of land use and seasonal climate variation (e.g. precipitation) on the spatial and temporal biodiversity and relative abundance of mosquitoes on St. Kitts. Below are a description of our methods and our findings.

## Methods

### Study area

**Fig. 1:**
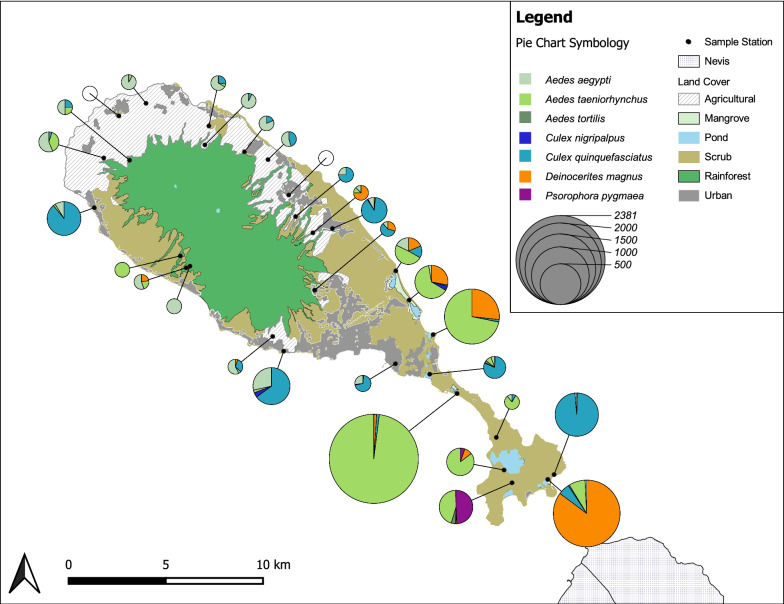
Numbers and species of mosquitoes trapped on St. Kitts at 30 sites comprising six replicates in each of five land covers

St. Kitts (Fig. 1) is a 168 km^2^, geographically isolated, volcanic, Caribbean island located in the Lesser Antilles (17.33°N, 62.75°W). It has a population of approximately 40,000 people mostly inhabiting Basseterre, the capital, and a string of small village communities distributed along the main coastal road which circles the island. The climate in St. Kitts is tropical, driven by constant sea breezes with little seasonal temperature variation (27–30 °C). The wet season runs from May to November with risk of hurricanes from June to November. Rainforest covers the uninhabited, steep volcanic slopes in the center of the island, surrounded by lower gentler slopes consisting mostly of abandoned sugar cane fields or arable farmlands. The south east of the island is primarily an arid peninsula covered mainly in scrub with beaches, mangroves, and salt-ponds.

### Mosquito sampling

To estimate the diversity and relative abundance of mosquito species across different land covers and seasons, we evaluated counts and species identity of adult mosquitoes captured in trap arrays set across the island. Trapping was carried out monthly from November 2017 to March 2019 in each of five representative land covers unless there was inclement weather. Due to high spatial heterogeneity in potential habitat, we used a randomized simplified stratified sampling design [13, 14] to increase the precision of generating a representative sample of the mosquito community on St. Kitts. To do this, we stratified St. Kitts by the five common land uses on the island (agricultural, mangrove, rainforest and urban). We then created a grid of St. Kitts and randomly, when possible, selected six replicate sites at least 1 km apart in each of these five distinct land cover categories producing 30 sites in total (Fig. 1). Final site selection was ultimately dependent on accessibility and landowner consent.

We used Biogents Sentinel 2 traps (BGS) (Biogents AG, Regensburg, Germany) baited with the BG-sentinel lure (Biogents AG, Germany) and carbon dioxide CO_2_. Carbon dioxide was generated by mixing 35 g of dried bread making yeast (Fleischmannʼs Active Dry Yeast, USA), 0.7 kg of unbranded white sugar, and approximately 2.5 l water in a 5 l water bottle. Carbon dioxide was delivered to each trap *via* a 5 m length of 5 mm (internal diameter) PVC tubing [15–17]. Although yeast generated CO_2_ will collect significantly fewer mosquitoes than dry ice or compressed CO_2_ from cylinders, it provides a useful alternative that is cheaper and more easily obtained than dry ice in tropical areas [15, 17, 18]. Traps were run monthly during the study period when possible for 48 h, with yeast-sugar solution, batteries, and catch bags replaced every 24 h. Trapped mosquitoes were transported to the research laboratory of Ross University School of Veterinary Medicine (RUSVM) and stored at -80 °C for later identification. After being rehydrated on chilled damp tissue paper, mosquitoes were identified on a chill table using morphological keys under a stereomicroscope (Cole Palmer, USA) at 10–40× magnification [19–21]. Counts of each mosquito species were recorded for each sampling date, land use, and location.

### Estimating mosquito diversity

We used a hierarchical Bayesian parameterization of the multi-species occupancy model (MSOM) of Royle & Dorazio [22] and Dorazio et al. [23] with data augmentation [24] to estimate true species diversity and its variation among our surveyed sites, while accounting for inter-species heterogeneity in detection:

$$R=n+{\sum }_{i=1}^{{n}_{aug}}{w}_{n+i}$$

$${w}_{i} \sim \mathrm{Bernoulli}(\Omega )$$

$${Z}_{j,i} \sim \mathrm{Bernoulli}({\psi }_{j,i}\times {w}_{i} )$$

$${X}_{j,k,i} \sim \mathrm{Bernoulli}({p}_{j,k,i}\times {Z}_{j,i}),$$

where $R$ is the posterior distribution of simulated species richness, $n$ is the number of of observed species, ${n}_{aug}$ is the number of augmented (all-zero capture history) species added to the dataset, $Z$ is the latent occupancy variable, and $X$ is the data. Species occurrence (${\psi }_{j,i}$) and detection (${p}_{j,k,i}$) were modeled as hierarchical random effects,

$$\mathrm{logit}\left({p}_{j,k,i}\right)={v}_{i}$$

$${v}_{i}\sim \mathrm{Normal}({\mu }_{v},{\tau }_{v})$$

$$\mathrm{logit}({\psi }_{j,i})={u}_{i}$$

$${u}_{i}\sim \mathrm{Normal}({\mu }_{u},{\tau }_{u})$$

We used weakly informative Gaussian priors for ${\mu }_{u}$ and ${\mu }_{v}$ with a mean of 0 and a standard deviation of 2.25 [25, 26] and vague gamma ($r$ = 0.1, $\lambda$= 0.1) priors for their precision, ${\tau }_{u}$ and ${\tau }_{v}$. Omega was given a flat uniform (0, 1) prior. We also monitored derived measures of diversity: alpha-diversity (*α*, mean site-level species richness) and beta-diversity (*β*, ratio between regional and site-level species richness) [27], zeta-diversity (*ζ*, number of species present at all sites) [28], and the number of sites each species occupied. We fit our model in JAGS 4.3.0 [29] implemented in the program R [30] using the R package *runJAGS* [31]. Posterior parameter estimates were drawn from three 20,000-iteration MCMC chains following a 1000-iteration adaptation period, and 10,000 iterations of burn-in. Convergence was assessed using the $\widehat{R}$ statistic [32] and by visually inspecting and comparing each MCMC chain’s sample traces and posterior sampling distributions. We illustrated our overall diversity results by plotting the posterior median and 90% credible interval of our diversity metrics of interest (*R*, *α*, *β*, and *ζ*). We then illustrated among-site variation in species diversity with respect to percent local land cover by plotting the posterior median and 90% credible intervals of site-level richness estimates against site-level proportion of local land covers: scrub, agriculture, mangrove, rainforest and urban.

### Estimating relative mosquito abundance

We evaluated influences of different land covers (agricultural, mangrove, rainforest, scrub, urban) on the relative abundance of the four most common mosquito species found in our survey. The land covers in a 1 km^2^ area (565 m radius) around each sampling site were determined from local observation, a remote sensing vegetation classification [33], the St. Christopher (St. Kitts) and Nevis Biodiversity Strategy and Action Plan [34], and the most recent Google Imagery (2019). When discordance in ascribing land covers was found between the different methods, the Google images were used preferentially. The percentages of each land cover at each site (Additional file 1: Figure S1) were calculated and used as a continuous covariate in establishing the models.

We assessed the effects of land cover at each trap location and monthly precipitation on the numbers of the four most common mosquito species trapped using mixed effects generalized linear regression models [30]. Our response variable for this analysis was the number of mosquitoes of each species captured by BGS traps during each 48-h trapping interval. A list of variables and general expectations of their effects on the counts of mosquitoes of each species captured can be found in Table 1. We also included two categorical variables reflecting the high affinity of some mosquito species (e.g. *Ae. taeniorhynchus* and *De. magnus*) for mangrove habitat and crabhole habitat located in the vicinity of mangroves [11, 35–37]. These variables included “mangrove”, which described the land cover of sites that fell within mangrove habitats regardless of surrounding land covers, and “m_trait”, which described a mosquito species preference for mangrove habitat. Monthly precipitation measurements were obtained at the Robert L. Bradshaw International Airport and accessed as archived data downloaded from the Weather Underground website (www.wunderground.com: accessed August 2019).

**Table 1:** Variables and associated hypotheses evaluated in statistical models

Variable	Explanation	Hypothesis
Precip	The sum of rainfall for the month measured at the Bradshaw International Airport	Increased rainfall is associated with higher mosquito abundance as desiccation risk of adults is reduced and viable oviposition and larval rearing sites are more abundant during periods of higher rainfall
m_trait	Species-specific breeding specialization in mangrove	If a species has a mangrove breeding specialization, it is associated with higher abundances within that land cover
LocalAgricultural	Percentage agricultural land cover in the 1 km area surrounding sampling sites	Nearby agricultural land cover is associated with lower abundance of non-anthrophilic mosquito species
LocalMangrove	Percentage mangrove land cover in 1 km area surrounding sampling sites	Nearby mangrove land cover is associated with higher abundances of mangrove mosquitoes
LocalRainforest	Percentage of rainforest land cover in 1 km area surrounding sampling sites	Nearby rainforest land cover is associated with lower abundance of mosquitoes that are associated with mangrove or anthropogenic habitats
LocalUrban	Percentage of urban land cover in 1 km area surrounding sampling sites	Nearby urban land cover is associated with higher abundances of anthrophilic mosquitoes and lower abundances of others
LocalScrub	Percentage of scrub land cover in 1 km area surrounding sampling sites	Nearby scrub land cover is associated with lower mosquito abundance
LocalAnthropogenic	The sum of urban and agricultural land cover	Local anthrophogenic land cover is associated with higher abundances of anthrophilic mosquitoes and lower abundances of others
Mangrove	A categorical variable indicating that the sampling site is located within the mangrove land cover	Mangrove land cover is associated with higher mangrove mosquito abundance within mangrove habitat

We fitted models to predict observed counts of the four most abundant mosquitoes in our dataset using spatial variation in landscape variables and seasonal variation in precipitation using the R package *glmmTMB*, which allows the specification of generalized linear mixed-effects models for a variety of error distributions, including Poisson and negative binomial distributions [38]. Preliminary analyses revealed that a negative binomial distribution with a quadratic variance-to-mean relationship best explained our data [39] (Additional file 2: Table S1), and we used this error distribution for all subsequent analyses. We assumed a linear relationship between overall mosquito counts (on the log-link scale) and monthly average precipitation at the island scale to account for intra-annual seasonality (e.g. wet *vs* dry seasons). Species-specific random slope and intercept terms for precipitation allow its effect to vary by species, and random site intercepts account for repeat observations at each site. The effects of land cover were allowed to vary independently by species. We evaluated 12 model hypotheses of species-specific variation in relative abundance with land cover. Land cover variables incorporated in our 12 models generally include the percentage of local land cover. We excluded any hypotheses for which variance inflation factors were greater than five prior to model evaluation and used AIC to select our best model from the candidate set [40]. To assess model fit, we evaluated the distribution of re-scaled model residuals from the R package *DHARMa* [41] and calculated conditional and marginal *R*^2^ values following Nakagawa et al. [42]. We used site-level predictions from our best model to show model-estimated trends in mosquito abundance across land use and season for the duration of our study.

## Results

From November 2017 to March 2019 we captured 10 of the 14 species previously recorded on St. Kitts [11, 12, 19] (Fig. 1, Additional file 3: Table S2 and Additional file 4: Table S3). We were unable to trap during the months of April and December 2018 due to inclement weather events. Voucher specimens were deposited in the United States National Museum (USNM) under the following catalog numbers USNMENT01239050-74 and USNMENT01239079. The most abundant species of mosquito was *Aedes taeniorhynchus* (*n* = 3861, mean = 276, SD = 643), which was primarily found in mangroves (88.4%). *Culex quinquefasciatus* (*n* = 1663, mean=119, SD = 121) was the second most abundant species primarily captured in urban areas (48.8%). *Deinocerites magnus* (*n* = 1577, mean = 113, SD = 150) and *Aedes aegypti* (*n* = 443, mean = 32, SD = 40) were the third and fourth most abundant mosquito species captured, respectively. *Aedes aegypti* (*n* = 443, mean = 89, SD = 161), *Ae. taeniorhynchus* (*n* = 3861, mean = 772, SD = 1830), *Cx. quinquefasciatus* (*n* = 1663, mean = 333, SD = 628), and *Deinocerites magnus* (*n* = 1577, mean = 315, SD = 797) were species captured in all five land covers. All other species were much less abundant. *Psorophora pygmaea* and *Toxorhynchites guadeloupensis* were only captured in scrub habitat, with the remaining species being distributed across more than one land cover. The highest overall number of mosquitoes captured, mostly *Ae. taeniorhynchus*, were caught in November 2018 (*n* = 3786), and monthly mean average catches were higher in general during the wet season (*n* = 1080) than the dry season (*n* = 177). *Aedes aegypti* was the only species to be captured during every trapping month. Only four *Anopheles albimanus*, the main vector of malaria in the Caribbean [43], were caught across the entire survey period. Finally, due to specimen damage during capture and transport to the laboratory, a small proportion of *Aedes* spp. (*n* = 305) and a larger portion of *Culex* spp. (*n* = 1687) were only reported to the genus level (Additional file 3: Table S2 and Additional file 4: Table S3).

### Mosquito community diversity

**Fig. 2:**
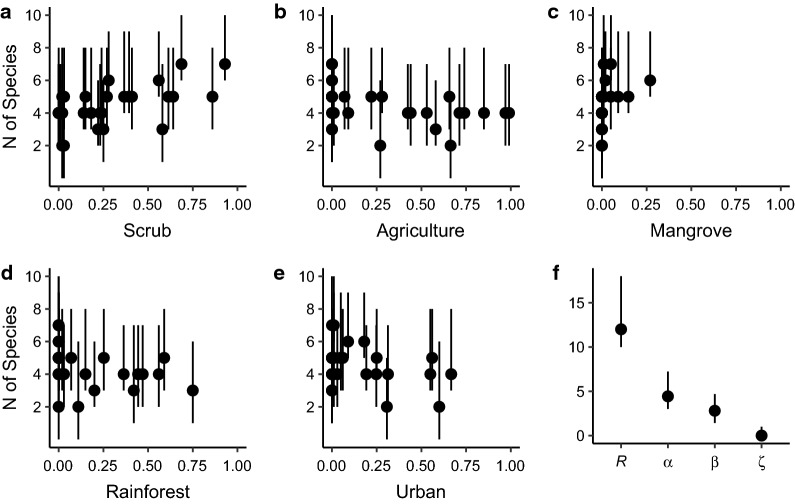
Median values for species richness and 90% credible intervals for each site from our multi-species occupancy model are plotted against the percentage of scrub forest (**a**), agriculture (**b**), mangrove (**c**), rainforest (**d**), and urban (**e**) land covers. **f** Median values and 90% credible intervals of regional species richness (*R*), alpha-diversity (*α*), beta-diversity (*β*), and zeta-diversity (*ζ*)

Our diversity analysis predicted that there are more mosquito species on St. Kitts than we directly observed, but by a reasonably low margin. We observed 10 species in our survey, while our model indicates that true species richness (*R*) on St. Kitts falls within 10–18 species (90% Bayesian credible interval), with a median of 13 species (Table 2). The logit-mean occupancy (*µ*_*u*_) and detectability (*µ*_*v*_) parameters indicated a mean occupancy probability of 31% and a mean detection probability of 9% for species present on the island (Table 2). We also predicted the number of species per site (alpha-diversity, α) to be 3–7 species (median: 4 species), the change in diversity of species among sites (beta-diversity, *β*) to be 1–5 species (median: 3 species), and the number of species present at all sites (zeta-diversity, *ζ*) to range from 0–1 species (median: 0 species) (Table 2, Fig. 2). Two highly abundant species (*Ae. aegypti* and *Cx. quinquefasciatus*), that are also important disease vectors, had relatively high predicted occupancy across our 30 study sites (*Ae. aegypti*: 26 sites; *Cx. quinquefasciatus*: 22 sites; Table 2). Finally, we observed potential effects of local land cover on overall species richness, with the overall richness increasing in response to the percentage of scrub and mangrove land cover and decreasing with the percentage of agricultural and urban land cover (Fig. 2).

**Table 2:** Median values and 90% credible interval for parameter estimates, mosquito community diversity metrics, and the number of sites predicted to have *Aedes aegypti* and *Culex quinquefasciatus* from our multiple species occupancy model

Parameter	Median	5% CI	95% CI
*Ω*	0.64	0.37	0.94
*µ*_*u*_	− 0.73	− 2.48	0.88
*σ*_*u*_	2.03	0.52	4.29
*µ*_*v*_	− 2.30	− 3.77	− 1.12
*σ*_*v*_	1.68	0.40	3.58
*R*	13.00	10.00	18.00
*α*	4.50	2.97	7.23
*β*	2.80	1.49	4.60
*ζ*	0.00	0.00	1.00
No. of sites *Ae. aegypti*	26.00	24.00	30.00
No. of sites *Cx*. *quinquefasciatus*	22.00	21.00	24.00

### Mosquito abundance is affected by land cover and seasonal precipitation

The best model of temporal variation in the relative abundance of the four most common mosquito species on the Island of St. Kitts was the model H12 from Table 3. This model included the effects of monthly precipitation, a mangrove breeding trait interaction with mangrove sites, and species-specific effects driven by agriculture, urban, and rainforest land covers. Our best model can be expressed in pseudo-code as

**Table 3:** A list of main effects of all candidate models considered in analyses of land cover effects on mosquito relative abundance

Name	Main effects	ΔAIC	K
H1	Precip + LocalAgriculture + LocalUrban + LocalRainforest + LocalMangrove + spp*Mangrove	11.4	26
H2	Precip + LocalAnthropogenic	53.6	11
H3	Precip + LocalAgriculture + LocalUrban	31	15
H4	Precip + LocalAnthropogenic + m_trait**:**Mangrove	38.6	14
H5	Precip + LocalAgriculture + LocalUrban + m_trait**:**Mangrove	14.5	18
H6	Precip + LocalScrub+ LocalAnthropogenic + m_trait**:**Mangrove	37.2	18
H7	Precip + LocalAgriculture + LocalUrban + LocalScrub+ m_trait**:**Mangrove	15	22
H8	Precip + LocalRainforest + LocalAnthropogenic + m_trait**:**Mangrove	25.6	18
H9	Precip + LocalAgriculture + LocalUrban + LocalRainforest + m_trait**:**Mangrove	3.9	20
H10	Precip + LocalRainforest + LocalScrub+ LocalAnthropogenic + m_trait**:**Mangrove	26.8	22
H11	Precip + LocalAgriculture + LocalUrban + LocalRainforest + LocalScrub+ m_trait**:**Mangrove	3.9	26
H12	Precip + LocalAgriculture + LocalUrban + LocalRainforest + m_trait*Mangrove	0	22

*Notes*: Models were ranked in terms of the difference between a given AIC score and that with the lowest AIC score (minAIC): ΔAIC=AIC-min(AIC). K represents the number of parameters in each model

**Fig. 3:**
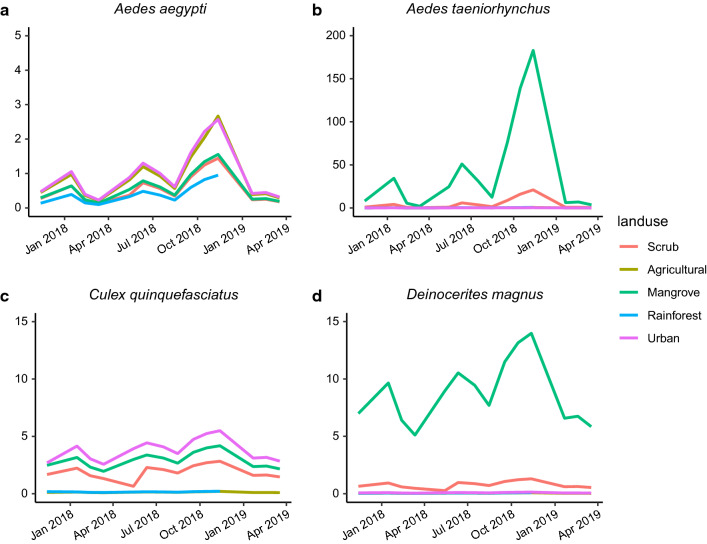
Time series plot of predicted relative abundance (conditional on random effects) from our best model for each site land cover (line color) and for the four major species we captured (panel). Solid lines denote the average predicted relative abundance of mosquito species across the six sites located within that land cover

**Fig. 4:**
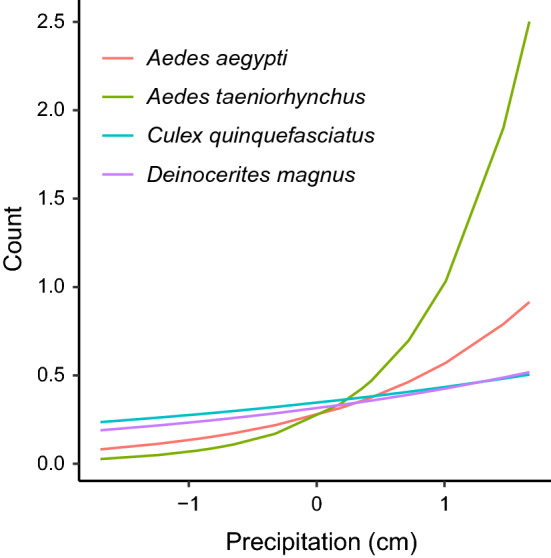
Best linear unbiased predictors (BLUPs) for the effect of precipitation on mosquito counts

*Two variables with full factorial interaction comprised of main effects and interaction terms; “**:**”, cases where only the interaction term is evaluated

1 $$\begin{aligned}
y_{(i,j,s)} &\sim \mathrm{NegBin}(\mu_{(i,j,s)}, \theta) \\
\mathrm{log}({\mu_{(i,j,s)}}) &= \beta_{0} + \alpha_{0(s)} + (\beta_{1} + \alpha_{1(s)})Precip_{(j)} + \beta_{2}m_{trait(s)} + \beta_{3} Mangrove_{(i)} + \beta_{4(s)}LocalAgriculture_{(i)} + \beta_{5(s)}LocalUrban_{(i)} + \beta_{6(s)}LocalRainforest_{(i)} + \beta_{7}m_{trait(s)}Mangrove_{(i)} + \alpha_{2(i)}
\end{aligned}$$

where $i$, $j$, and $s$ denote indices for site, month, and species, respectively. $\mathrm{NegBin}$ reflects the negative binomial distribution, and the final model includes the following: random intercepts for each species (${\beta }_{0}+{\alpha }_{0(s)}$), a main effect of precipitation with species-specific random slopes ($({\beta }_{1}+{\alpha }_{1(s)})Preci{p}_{(j)}$), an interaction between mangrove and the mangrove breeding trait (${{{\beta }_{2}{m\_trait}_{(s)}+\beta }_{3}Mangrov{e}_{(i)}+\beta }_{7}{m\_trait}_{(s)}Mangrov{e}_{(i)}$), species-specific main effects on proportional local land cover variables (${{\beta }_{4(s)}LocalAgricultur{e}_{(i)}+\beta }_{5(s)}LocalUrba{n}_{(i)}+{\beta }_{6(s)}LocalRainfores{t}_{(i)}$), and a site-level random intercept term (${\alpha }_{2(i)}$). The variance for our best negative binomial model scales quadratically with the mean ($\mu$): $Var({y}_{(i,j,s)})={\mu }_{(i,j,s)}(1+\frac{{\mu }_{(i,j,s)}}{\phi })$ [39]. Our best model’s predicted relative abundance for each mosquito species, averaged across each site’s land cover, illustrated species-specific responses to surrounding land cover and seasonal precipitation effects across the island (Table 4, Fig. 3, Additional file 5: Figure S2). Overall, we observed a positive effect of precipitation on the relative abundance of mosquitoes (${\beta }_{1}$), with significant among-species variation in this relationship (${\alpha }_{1(s)}$, Table 4). To further explore the effect of precipitation, we derived the best linear unbiased predictors (BLUPs) for each species’ precipitation effect. The BLUPs suggested that the effect of precipitation was strongest for *Ae. taeniorhynchus* and to a lesser extent *Ae. aegypti* (Fig. 4), and was weakest for *Cx. quinquefasciatus* and *De. magnus*. The model also predicted significantly negative relationships between urban land cover and *Ae. taeniorhynchus* and *De. magnus*, but only slightly positive, non-significant relationships between urban land cover and the urban-associated mosquitoes, *Cx. quinquefasciatus* and *Ae. aegypti*. Rainforest and agricultural land cover were negatively associated with the relative abundance of all species we considered except *Ae. aegypti*, which exhibited no significant covariation with either rainforest or agricultural land cover (Table 4). As expected, the model predicts the relative abundance of mosquito species with a mangrove breeding preference (*Ae. taeniorhynchus* and *De. magnus*) to be lower than average except when trapping sites occur within mangrove habitat where the mangrove “trait” had a net positive effect (i.e. $({\beta }_{2}+{\beta }_{7})>0$, Table 4). Inspection of re-scaled residuals generated by simulation from the fitted model [41] suggested uniformity in the distribution of residuals (one-sample Kolmogorov-Smirnov test: *D* = 0.018, *P* = 0.671), indicating good concurrence between the data and model predictions. Marginal and conditional *R*^2^ (0.601 and 0.696, respectively) also indicated a well-fit model (Table 4): the marginal *R*^2^ of 0.601 suggested that the fixed effects portion of the model explained over 60% of the variation in counts, and the conditional *R*^2^ of 0.695 revealed the additional variance explained by accounting for additional variation attributable to the random effects [42].

**Table 4:** Model parameters and diagnostics from our best model of mosquito counts. Means and confidence intervals are given on the log-scale, and symbols correspond to parameters in equation 1

Predictors	Symbol	Mean	95 % CI	P
Intercept	${\beta }_{0}$	− 0.75	− 1.33– − 0.18	0.01
Precipitation	${\beta }_{1}$	0.66	0.13–1.19	0.015
Mangrove Breeding Trait	${\beta }_{2}$	− 1.4	− 2.12– − 0.69	< 0.001
Mangrove site category	${\beta }_{3}$	0.01	− 1.29–1.31	0.99
Local Agriculture (*Ae.ae*.)	${\beta }_{4(Ae. ae.)}$	0.2	− 0.38–0.77	0.503
Local Agriculture (*Ae.ta*.)	${\beta }_{4(Ae.ta.)}$	− 1.35	− 1.98– − 0.71	< 0.001
Local Agriculture (*Cx.qu*.)	${\beta }_{4(Cu.qu.)}$	− 1.07	− 1.65– − 0.49	< 0.001
Local Agriculture (*De.ma*.)	${\beta }_{4(De.ma.)}$	− 1.08	− 1.80– − 0.35	0.003
Local Urban (*Ae.ae*.)	${\beta }_{5(Ae. ae.)}$	0.13	− 0.42–0.68	0.644
Local Urban (*Ae.ta*.)	${\beta }_{5(Ae.ta.)}$	− 2.11	− 2.91– − 1.30	< 0.001
Local Urban (*Cx.qu*.)	${\beta }_{5(Cu.qu.)}$	0.32	− 0.22–0.85	0.248
Local Urban (*De.ma*.)	${\beta }_{5(De.ma.)}$	− 0.97	− 1.67– − 0.26	0.007
Local Rainforest (*Ae.ae*.)	${\beta }_{5(Ae. ae.)}$	− 0.43	− 0.99–0.13	0.129
Local Rainforest (*Ae.ta*.)	${\beta }_{5(Ae.ta.)}$	− 1.21	− 1.94– − 0.48	0.001
Local Rainforest (*Cx.qu*.)	${\beta }_{5(Cu.qu.)}$	− 1.39	− 2.03– − 0.76	< 0.001
Local Rainforest (*De.ma*.)	${\beta }_{5(De.ma)}$	− 0.91	− 1.64– − 0.18	0.014
Mangrove Breeding × Mangrove Site	${\beta }_{7}$	2.32	0.83–3.81	0.002
Overdispersion	$\phi$	0.0962		
Random effects				
Site Intercept	${\alpha }_{2(i)}$	0.54		
Species Intercept	${\alpha }_{0(s)}$	0.02		
Species × Precipitation Slope	${\alpha }_{1(s)}$	0.24		
Correlation of Species random effects		− 0.69		
Number of sites		30		
Number of species		4		
Observations		1568		
Marginal *R*^2^ / Conditional *R*^2^		0.601/0.695		

*Abbreviations: Ae.ae*., *Ae. aegypti*; *Ae.ta*., *Ae. taeniorhynchus*; *Cx.qu.*, *Cx. quinquefasciatus*; *De.ma.*, *De. Magnus*

## Discussion

Monthly mosquito surveillance on St. Kitts from November 2017 to March 2019 enabled us to capture a diversity of mosquito species that varied in abundance across seasons and land cover types. We captured 10 of the 14 species (5 genera) historically recorded on the island [11, 12, 19]. While our results largely confirm those of the 2010 survey, they provide higher spatial and temporal resolution of the mosquito community diversity, as well as the relative abundance and distribution of different mosquito species on the island.

Our multi-species occupancy model demonstrates that we were able to capture most mosquito species on St. Kitts during the survey period and that any detection failures in other mosquito species on the island are likely attributed to low rates of occurrence. Thus, the species detected in our survey likely are an accurate reflection of the true mosquito community on St. Kitts. While we were able to detect the majority of species predicted to be present on St. Kitts, our actual ability to detect a given mosquito species was low on average (9% average detection rate). That being said, multi-species occupancy models are robust to low detection probabilities as long as mean site-level occupancy is relatively high, which it was in this study (30%) [44]. Using the site-level species richness estimated from our multi-species occupancy model, we noted some potential correlation between percentage of local land cover and mosquito species richness. However, our values for beta- and zeta-diversity indicate species turnover across sites and few to no species found everywhere. The effects of land use on site-level species richness may be masked by species replacement driven by species-specific responses to landscape variables, which warrants further investigation into species-specific responses to landscape variation.

The four most abundant species captured were *Ae. taeniorhynchus*, *Cx. quinquefasciatus*, *Ae. aegypti* and *De. magnus*. These species were detected at least once in all land cover types over the study period. The three most abundant species captured are competent vectors of pathogens recorded on St. Kitts (*Cx. quinquefasciatus*: West Nile virus and *Dirofilaria immitis*; *Ae. taeniorhynchus*: *D. immitis*; and *Ae. aegypti*: dengue, chikungunya and Zika viruses) [45–49] and other pathogens that could potentially become established on the island in the future due to the abundance of their vectors. For example, *Cx. quinquefasciatus*, the southern house mosquito, is widely distributed across the subtropics and can also transmit Saint Louis encephalitis virus, Western equine encephalitis virus, Rift Valley fever virus, *Wuchereria bancrofti* and avian malaria [21]. *Aedes taeniorhynchus*, the black salt marsh mosquito, is widely distributed in all islands of the Caribbean and can transmit Venezuelan, Eastern and Western equine encephalitis virus [50, 51]. The eponymous yellow fever mosquito, *Ae. aegypti*, can also transmit yellow fever virus [21]. While *An. albimanus* is a known malaria vector in Central America, northern South America, and the Caribbean, the overall low abundance of this species on St. Kitts (only four individuals total were collected in our study and two individuals in 2010 [12]), suggests this species is unlikely to support malaria transmission on the island.

The presence and absence, as well as overall relative abundance, of particular mosquito species captured across the different land covers on St. Kitts broadly align with what is known for these species in the literature. The species count model for our four most abundant mosquitoes predicts both *Cx. quinquefasciatus* and *Ae. aegypti* to have the highest relative abundance in an urban habitat. Both species breed most successfully in fresh water-filled man-made containers and are therefore found primarily around houses in urban environments. Further, *Ae. aegypti* preferentially feeds on human hosts, particularly when indoors [21, 52] and rests inside domestic dwellings [21]. The fact that we observed these species, albeit at lower abundances, in other land covers is not entirely surprising. The model predicted *Ae. aegypti* to be similarly abundant across the survey period in urban as well as agricultural habitats. From local experience and surveys on other islands [53], agricultural land covers provide ample breeding habitats for container breeding mosquito species in the form of discarded tires, styrofoam containers, plastic water bottles and bags, and agricultural equipment in which water can collect.

Interestingly, the model also predicted moderate relative abundance across the survey period for both *Cx. quinquefasciatus* and *Ae. aegypti* in mangrove and scrub habitats, which may be due to the presence of artificial containers suitable for breeding or an increased tolerance to brackish water in high marsh areas of the mangrove [36, 53]. *Culex quinquefasciatus* has been recorded previously in brackish water on St. Kitts [11] and *Ae. aegypti* is tolerant of brackish water in other coastal regions [54–57]. Additionally, *Cx. quinquefasciatus* is an opportunistic forager that has the ability to fly several hundred meters [58]; thus, adults could be found in areas far removed from their larval breeding sites. Finally, *Ae. aegypti* was also predicted to be abundant, albeit at lower levels, in rainforest habitat across the trapping period. Elsewhere in the Caribbean, *Ae. aegypti* have been found breeding in more natural habitats in addition to artificial containers [37]. These include rock holes, calabashes, tree holes, leaf axils, bamboo joints, papaya stumps, coconut shells, bromeliads, ground pools, coral rock holes, crab holes, and conch shells which are all also present on St. Kitts.

Species with the highest capture rates and predicted by our model to have high relative abundance in mangrove habitats included both *Ae. taeniorhynchus* and *De. magnus*. These two species were predicted to have relatively low abundance in scrub surrounding mangrove sites on the island, and were not predicted to be abundant in other land cover types. *Aedes taeniorhynchus*, the black salt marsh mosquito, is widely distributed in all islands of the Caribbean where it also favors low lying marsh land as is the case on St. Kitts [36, 51]. Similarly, *De. magnus*, the crabhole mosquito, is found primarily in crabholes that are abundant in the soft sands in mangrove habitats around the Caribbean [11, 59]. We also captured *Culex nigripalpus*, *An. albimanus*, *Ps. pygmaea*, and *Aedes tortilis* in mangroves or the surrounding scrub land cover, likely due to their preference for breeding in temporary brackish and/or fresh water sources [11, 19]. We did not include these species in our relative abundance model due to low capture rates.

Generally, we had very low capture rates of mosquitoes in rainforest land cover which was consistent with our model’s predictions of a negative effect of rainforest cover on the relative abundance of the four most abundant species we trapped. This is not necessarily reflective of the results of surveys from other regions of the Caribbean. For example, a study in forested areas of eastern Trinidad between July 2007 and March 2009 collected 185,397 mosquitoes across 46 species [59]. Although this study was of a similar duration, our low capture rates in rainforest land cover might reflect (i) less breeding habitat or fewer vertebrate hosts present in the rainforest, (ii) different sampling methods across the studies (e.g. CDC light traps deployed with CO_2_ lures used vs. BGS traps baited with the human lure or CDC light traps baited with sugar-yeast CO_2_ lures [15]), and (iii) frequency of trapping effort (weekly *vs* monthly). It seems unlikely that our trapping at ground level may have excluded mosquito species that thrive in tree-top habitats because several arboviral surveillance studies in forests in Brazil [60], New Mexico [61], and other sites across the USA [62] demonstrate that various trapping methods (e.g. entomological nets, aspirators, and CDC light traps) set in the canopy did not catch significantly more mosquitoes than those on the ground. We did capture one *Aedes busckii* in the rainforest during our survey. A previous survey [11] as well as some experience with larval surveys of tree holes in rainforest habitats on St. Kitts (data not shown) suggest *Ae. busckii* could be a rain forest habitat specialist, but more data are needed. In general, little is known about the ecology of this mosquito other than that it is confined to the Lesser Antilles (Dominica, Grenada, Guadeloupe, Martinique, Montserrat, St. Kitts and Nevis and Saint Lucia) [63]. We also captured *Cx. quinquefasciatus*, *Ae. taeniorhynchus* and *De. magnus* at very low rates in the rainforest (Additional file 3: Table S2). We speculate that these captures could be the result of mosquitoes either breeding in man-made containers in neighboring agricultural habitat (*Cx. quinquefasciatus*) or mosquitoes being blown into novel habitats during tropical storms (*Ae. taeniorhynchus* and *De. magnus*). In the case of *De. magnus*, land crabs (*Gecarcinus ruricola*) do inhabit the rainforest, which could provide breeding and resting sites in their crabhole burrows for this specialist mosquito species [59].

Mosquito capture rates were also strongly determined by time of season and precipitation throughout the survey period. In general, our species count model predicted a positive effect of precipitation on the relative abundance of our four most common mosquito species. The effects of precipitation we found could be due to several reasons. Although excess rain may flush larvae from their habitats and decrease adult mosquito populations [64, 65], a seasonal increase in precipitation increases the abundance and persistence of larval habitats resulting in higher densities and overall capture rates [65–68]. Additionally, increased precipitation is associated with increased relative humidity, which has been shown to have important positive effects on the abundance [68–70], lifespan [70, 71], and activity and questing behavior [72, 73] of adult mosquitoes. Interestingly, the effect of precipitation on relative abundance was species-specific among the four most common mosquito species we found. Our *post-hoc* assessment of our model suggests the effect of precipitation had a strong effect on the relative abundances of *Ae. taeniorhynchus*, a moderate effect on *Ae. aegypti*, and smaller effects on *Cx. quinquefasciatus* and *De. magnus*. The strong effect of precipitation on *Ae. taeniorhynchus* might occur because this mosquito species relies largely on natural habitats, which are often dependent on local rainfall. While *Ae. aegypti* utilizes artificial, and human watered containers heavily for ovipositing, it is also known to oviposit in natural habitats on other Caribbean islands [37], which could become more abundant with increased rainfall. *Culex quinquefasciatus* might be less dependent on rainfall, as most individuals were captured in urban habitat and were most likely emerging from persistent, human-watered, artificial habitats. Whereas increased rainfall above a certain threshold might expand water bodies in mangroves, flooding crabholes that in turn could locally reduce breeding sites for *De. magnus* [58].

While the overall capture rates of *Ae. aegypti* were significantly lower across non-urban habitats, their presence in other land covers on St. Kitts and other Caribbean islands [11, 37] could have several implications for our understanding of the general ecology of this species and transmission of arboviruses in the Caribbean. Mosquitoes living across these land covers likely experience variation in local microclimate [69, 74], quality and quantity of oviposition sites [11, 37], and access to vertebrate species available for blood-feeding [75]. This variation in turn could result in potential disease transmission among sylvatic reservoirs (e.g. non-human primates) in some habitats and differential exposure of human populations to infectious mosquitoes on the island. We are currently conducting studies to confirm the presence of reproducing *Ae. aegypti* adults across each land cover and using blood-meal analysis and bait trapping to identify novel mosquito-host associations.

Our study provides a more comprehensive spatial and temporal (within-year) picture of the distribution of mosquito species on St. Kitts relative to previous surveys [11, 12, 19]. However, it suffers from several minor limitations. Due to specimen damage during capture and transport to the laboratory, a proportion of *Aedes* spp. (*n* = 219) and *Culex* spp. (*n* = 1694) were reported only to genus level. These counts differed substantially from those identified to species level, which comprised 4334 *Aedes* spp. and 1697 *Culex* spp. in total. Based on the capture location of these specimens, the majority of these individuals are likely *Ae. taeniorhynchus* (mangrove) and *Cx. quinquefasciatus* (urban), respectively. By not incorporating the unidentified mosquitoes into our relative abundance and diversity analyses, we are inherently assuming that each mosquito species has an equal chance of being unidentified. This assumption could be violated if the ability to identify specimens varies by species (e.g. species that tend to be captured at higher numbers may sustain more damage during trapping and handling), which could have implications for both our diversity and relative abundance models. For example, capture rates for *Ae. tortilis* and *Cx. nigripalpus* are likely underestimated. However, we believe these effects to be minimal. For the relative abundance model, we selected the four most abundant species for whom violation of this assumption would have a small chance of affecting the relative proportion of captured individuals. In the diversity analysis, we used a model that estimates variation in detectability by species, which will help account for violations of this assumption because it allows rarer species to be detected less often.

## Conclusions

Our island-wide mosquito survey has demonstrated that the species detected in our survey are a good representation of the mosquito community on St. Kitts. Further, the community of mosquitoes on the island is highly structured and likely shaped by local land cover. We also found substantial effects of land cover and seasonality (likely driven by variation in precipitation) on mosquito capture rates. Interesting insights gained from this study include the presence of *Ae. aegypti* in all the land covers we studied, which could have important implications for mosquito-borne disease transmission on the island. Further, human-adapted mosquito species (e.g. *Ae. aegypti* and *Cx. quinquefasciatus*) that are known vectors for many human relevant pathogens (e.g. chikungunya, dengue and Zika viruses in the case of *Ae. aegypti*; West Nile, Spondweni, Oropouche virus, and equine encephalitic viruses in the case of *Cx. quinqefasciatus*) are the most wide-spread (across land covers) and the least responsive to seasonal variation in precipitation. This somewhat counters the current literature suggesting *Ae. aegypti* is primarily found in highly urban habitats and feeds almost exclusively on human hosts. Finally, although *Aedes albopictus* occurs on other Caribbean islands [76], we did not find this species in our survey. Ongoing surveillance will be important to continue, as changes in land use and climate could lead to shifts in mosquito community composition, host contact rates, and mosquito-borne disease transmission in humans and animals.

## Supplementary information

**Additional file 1: Figure S1.** The proportions of the different land covers found in a 1 km^2^ area (565 m radius) around each of the trapping 30 sites used in the study.

**Additional file 2: Table S1.** Statistical and model methods.

**Additional file 3: Table S2.** Counts of mosquito species caught across the five different land covers from November 2017 to March 2019 on St. Kitts.

**Additional file 4: Table S3.** Counts of mosquito species per month on St. Kitts from Nov 2017 to March 2019 with the wet season highlighted in grey (May-November).

**Additional file 5: Figure S2.** Time series plot for each site land use category (column) and for each species (row) in their predicted relative abundance (conditional on random effects) from our best model. Solid lines denote the average predicted relative abundance of mosquito species across the six sites within that land cover and dotted lines denote the 95% confidence interval of that 6-site mean. Points denote the 6-site average relative abundance from the raw data. Note that the y-axis scale varies by species (row).

## Data Availability

The datasets supporting the conclusions of this article are included within the article and its additional files. Voucher specimens of some of the mosquito specimens associated with this study were deposited in the United States National Museum (USNM) under the following catalog numbers USNMENT01239050-74 and USNMENT01239079.

## Abbreviations

BGS: Biogents Sentinel 2 Trap
CO_2_: Carbon dioxide
CDC: Centre for Disease Control (USA)
RUSVM: Ross University School of Veterinary Medicine
USNM: United States National Museum
