# Supplementary figures and images for: Effects of seasonality and land use on the diversity, relative abundance, and distribution of mosquitoes on St. Kitts, West Indies
Source: Parasit Vectors. 2020 Nov 2;13:543. doi: 10.1186/s13071-020-04421-7 (PMC7607626; doi:10.1186/s13071-020-04421-7)

Proportion of Land Cover

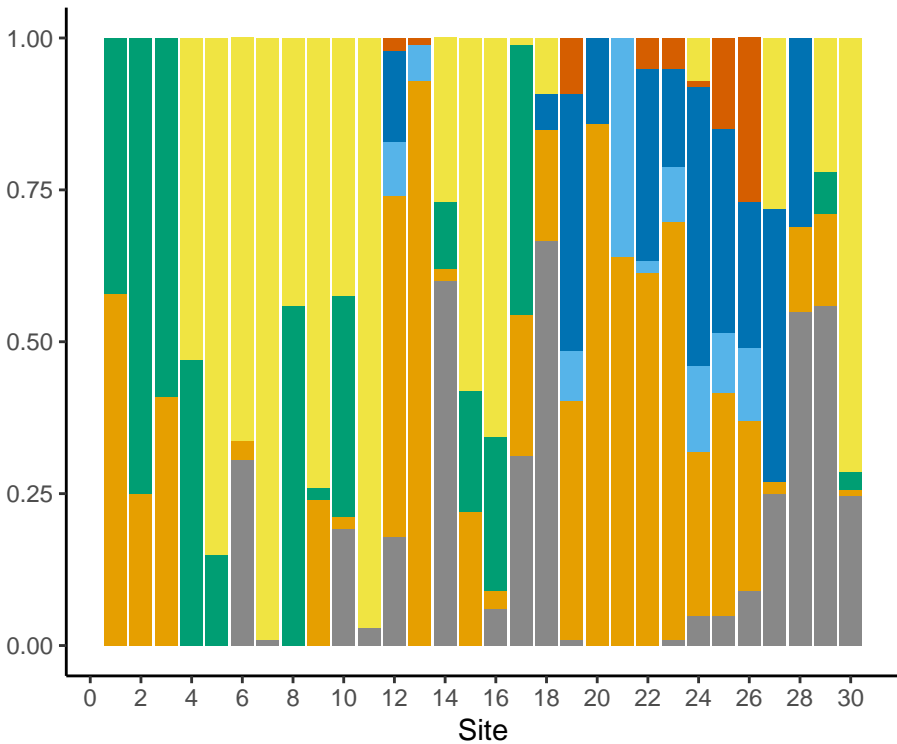

Land Cover Class

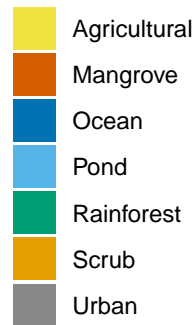

Supplement: Supplementary file 1 — Additional file 1: Figure S1. The proportions of the different land covers found in a 1 km2 area (565 m radius) around each of the trapping 30 sites used in the study. [file 13071_2020_4421_MOESM1_ESM.pdf]

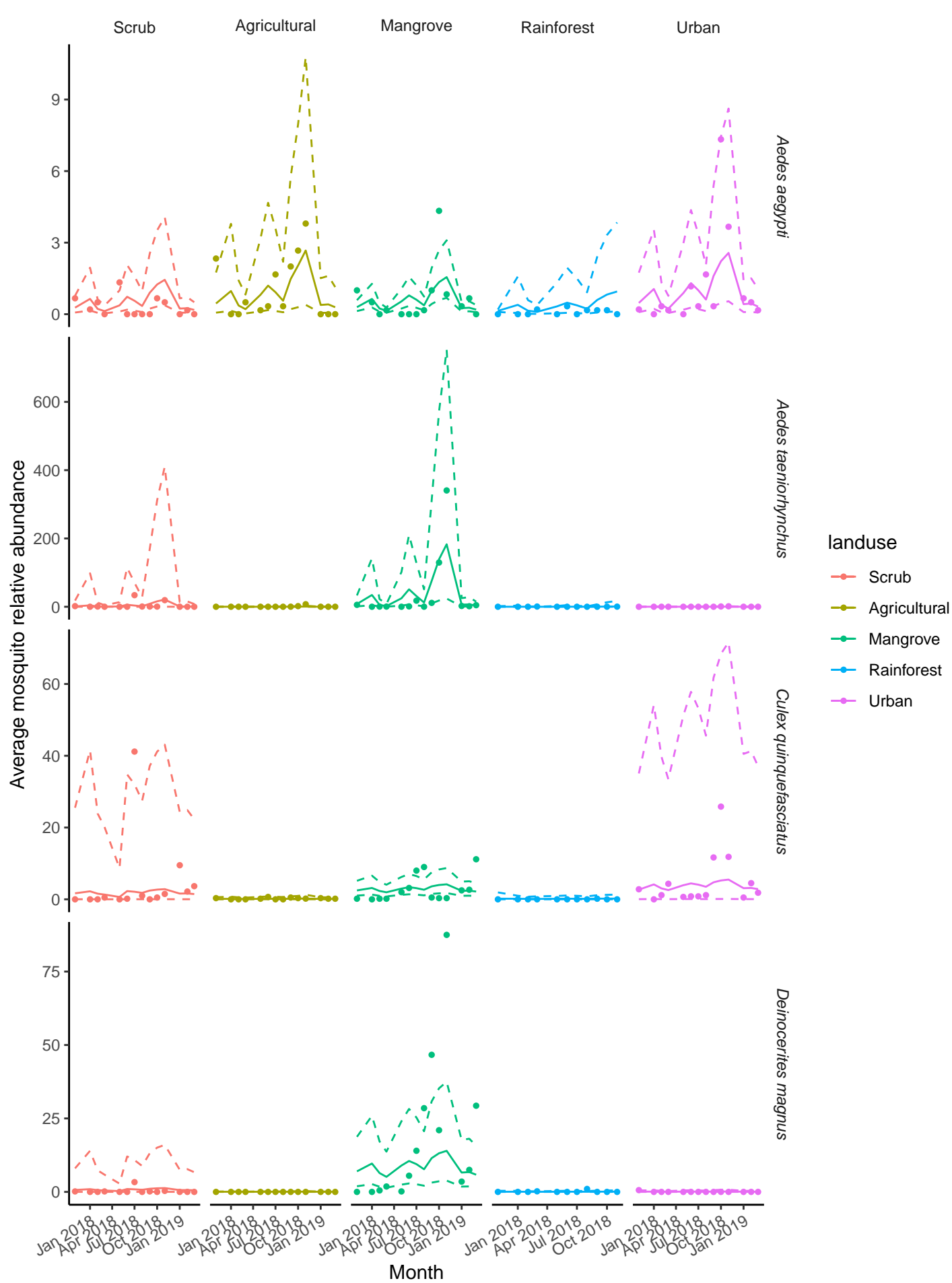

Supplement: Supplementary file 5 — Additional file 5: Figure S2. Time series plot for each site land use category (column) and for each species (row) in their predicted relative abundance (conditional on random effects) from our best model. Solid lines denote the average predicted relative abundance of mosquito species across the six sites within that land cover and dotted lines denote the 95% confidence interval of that 6-site mean. Points denote the 6-site average relative abundance from the raw data. Note that the y-axis scale varies by species (row). [file 13071_2020_4421_MOESM5_ESM.pdf]
