# Supplementary material for: Effects of seasonality and land use on the diversity, relative abundance, and distribution of mosquitoes on St. Kitts, West Indies
Source: Parasit Vectors. 2020 Nov 2;13:543. doi: 10.1186/s13071-020-04421-7 (PMC7607626; doi:10.1186/s13071-020-04421-7)
Supplement: Supplementary file 3 — Additional file 3: Table S2. Counts of mosquito species caught across the five different land covers from November 2017 to March 2019 on St. Kitts. [file 13071_2020_4421_MOESM3_ESM.docx]

**SI Table 2**: The total number of adults of each mosquito species caught across the five different land covers from November 2017 to March 2019 on St Kitts.

|  | Agricultural | Mangrove | Rainforest | Scrub | Urban | Grand Total | Mean | Standard Deviation |
| --- | --- | --- | --- | --- | --- | --- | --- | --- |
| *Aedes taeniorhynchus* | 50 | 3412 | 7 | 374 | 18 | 3861 | 772 | 1830 |
| *Aedes aegypti* | 154 | 67 | 11 | 25 | 186 | 443 | 89 | 161 |
| *Unidentified Aedes spp.* | 37 | 125 | 2 | 21 | 34 | 219 | 44 | 83 |
| *Aedes tortilis* | 0 | 14 | 0 | 14 | 0 | 28 | 6 | 11 |
| *Aedes busckii* | 1 | 0 | 1 | 0 | 0 | 2 | 0 | 1 |
| *Anopheles albimanus* | 1 | 3 | 0 | 0 | 0 | 4 | 1 | 2 |
| *Culex quinquefasciatus* | 23 | 261 | 1 | 566 | 812 | 1663 | 333 | 628 |
| *Unidentified Culex spp.* | 40 | 258 | 3 | 105 | 1288 | 1694 | 339 | 734 |
| *Culex nigripalpus* | 0 | 15 | 0 | 0 | 19 | 34 | 7 | 14 |
| *Culex bahamensis* | 0 | 0 | 0 | 0 | 0 | 0 | 0 | 0 |
| *Culex bisulcatus* | 0 | 0 | 0 | 0 | 0 | 0 | 0 | 0 |
| *Culex declarator* | 0 | 0 | 0 | 0 | 0 | 0 | 0 | 0 |
| *Culex madininensis* | 0 | 0 | 0 | 0 | 0 | 0 | 0 | 0 |
| *Deinocerites magnus* | 1 | 1533 | 8 | 26 | 9 | 1577 | 315 | 797 |
| *Psorophora pygmaea* | 0 | 0 | 0 | 178 | 0 | 178 | 36 | 92 |
| *Toxorhynchites guadeloupensis* | 0 | 0 | 0 | 1 | 0 | 1 | 0 | 1 |
| Grand Total | 307 | 5688 | 33 | 1310 | 2366 | 9704 | 1941 | 3772 |
