# Supplementary material for: Effects of seasonality and land use on the diversity, relative abundance, and distribution of mosquitoes on St. Kitts, West Indies
Source: Parasit Vectors. 2020 Nov 2;13:543. doi: 10.1186/s13071-020-04421-7 (PMC7607626; doi:10.1186/s13071-020-04421-7)
Supplement: Supplementary file 4 — Additional file 4: Table S3. Counts of mosquito species per month on St. Kitts from Nov 2017 to March 2019 with the wet season highlighted in grey (May-November). [file 13071_2020_4421_MOESM4_ESM.docx]

|  | Nov 13, 2017 | Jan 15, 2018 | Feb 12, 2018 | Mar 12, 2018 | May 14, 2018 | Jun 11, 2018 | Jul 16, 2018 | Aug 13, 2018 | Sep 17, 2018 | Oct 15, 2018 | Nov 12, 2018 | Jan 21, 2019 | Feb 18, 2019 | Mar 18, 2019 | Grand Total | Mean | Standard Deviation |
| --- | --- | --- | --- | --- | --- | --- | --- | --- | --- | --- | --- | --- | --- | --- | --- | --- | --- |
| *Aedes taeniorhynchus* | 63 | 89 | 18 | 6 | 0 | 9 | 321 | 5 | 111 | 811 | 2377 | 14 | 9 | 28 | 3861 | 276 | 643 |
| *Aedes aegypti* | 50 | 9 | 6 | 10 | 6 | 20 | 24 | 26 | 41 | 147 | 80 | 11 | 11 | 2 | 443 | 32 | 40 |
| Unidentified *Aedes* spp. | 71 | 24 | 0 | 1 | 9 | 4 | 10 | 9 | 34 | 41 | 16 | 0 | 0 | 0 | 219 | 16 | 21 |
| *Aedes tortilis* | 0 | 0 | 0 | 0 | 0 | 0 | 0 | 0 | 0 | 0 | 28 | 0 | 0 | 0 | 28 | 2 | 7 |
| *Aedes busckii* | 0 | 0 | 0 | 0 | 0 | 0 | 0 | 1 | 0 | 0 | 0 | 1 | 0 | 0 | 2 | 0 | 0 |
| *Anopheles albimanus* | 1 | 0 | 0 | 3 | 0 | 0 | 0 | 0 | 0 | 0 | 0 | 0 | 0 | 0 | 4 | 0 | 1 |
| *Culex quinquefasciatus* | 37 | 0 | 10 | 37 | 34 | 36 | 396 | 69 | 77 | 315 | 244 | 114 | 153 | 141 | 1663 | 119 | 121 |
| Unidentified *Culex* spp. | 210 | 19 | 21 | 63 | 9 | 22 | 27 | 98 | 360 | 544 | 318 | 3 | 0 | 0 | 1694 | 121 | 171 |
| *Culex nigripalpus* | 14 | 0 | 1 | 0 | 0 | 0 | 0 | 0 | 0 | 2 | 17 | 0 | 0 | 0 | 34 | 2 | 6 |
| *Culex bahamensis* | 0 | 0 | 0 | 0 | 0 | 0 | 0 | 0 | 0 | 0 | 0 | 0 | 0 | 0 | 0 | 0 | 0 |
| *Culex bisulcatus* | 0 | 0 | 0 | 0 | 0 | 0 | 0 | 0 | 0 | 0 | 0 | 0 | 0 | 0 | 0 | 0 | 0 |
| *Culex declarator* | 0 | 0 | 0 | 0 | 0 | 0 | 0 | 0 | 0 | 0 | 0 | 0 | 0 | 0 | 0 | 0 | 0 |
| *Culex madininensis* | 0 | 0 | 0 | 0 | 0 | 0 | 0 | 0 | 0 | 0 | 0 | 0 | 0 | 0 | 0 | 0 | 0 |
| *Deinocerites magnus* | 11 | 0 | 3 | 14 | 1 | 38 | 104 | 179 | 284 | 170 | 531 | 21 | 45 | 176 | 1577 | 113 | 150 |
| *Psorophora pygmaea* | 0 | 0 | 0 | 0 | 1 | 1 | 0 | 0 | 1 | 0 | 175 | 0 | 0 | 0 | 178 | 13 | 47 |
| *Toxorhynchites guadeloupensis* | 0 | 0 | 0 | 1 | 0 | 0 | 0 | 0 | 0 | 0 | 0 | 0 | 0 | 0 | 1 | 0 | 0 |
| Grand Total | 457 | 141 | 59 | 135 | 60 | 130 | 882 | 387 | 908 | 2030 | 3786 | 164 | 218 | 347 | 9704 | 693 | 1036 |

**SI Table 3:** The total number of adult mosquitoes of each mosquito species per month on St Kitts from Nov 2017 to March 2019 with the wet season highlighted in grey (May-November).
